# Seeing more than the Tip of the Iceberg: Approaches to Subthreshold Effects in Functional Magnetic Resonance Imaging of the Brain

**DOI:** 10.1007/s00062-024-01422-2

**Published:** 2024-06-06

**Authors:** Benedikt Sundermann, Bettina Pfleiderer, Anke McLeod, Christian Mathys

**Affiliations:** 1 Institute of Radiology and Neuroradiology, Evangelisches Krankenhaus Oldenburg, Universitätsmedizin Oldenburg, Steinweg 13–17, 26122 Oldenburg, Germany; 2 Research Center Neurosensory Science, Carl von Ossietzky Universität Oldenburg, Oldenburg, Germany; 3 Clinic of Radiology, Medical Faculty, University of Münster, Münster, Germany

**Keywords:** Functional magnetic resonance imaging, Statistics, Sub-threshold effects, Data visualization, Neuroimaging

## Abstract

Many functional magnetic resonance imaging (fMRI) studies and presurgical mapping applications rely on mass-univariate inference with subsequent multiple comparison correction. Statistical results are frequently visualized as thresholded statistical maps. This approach has inherent limitations including the risk of drawing overly-selective conclusions based only on selective results passing such thresholds. This article gives an overview of both established and newly emerging scientific approaches to supplement such conventional analyses by incorporating information about subthreshold effects with the aim to improve interpretation of findings or leverage a wider array of information. Topics covered include neuroimaging data visualization, *p*-value histogram analysis and the related Higher Criticism approach for detecting rare and weak effects. Further examples from multivariate analyses and dedicated Bayesian approaches are provided.

## Introduction

Parametric mapping of results of a high number of univariate statistical tests [1] has been a hallmark of functional magnetic resonance imaging (fMRI) analyses. Implemented in major software packages [2–4] it is in widespread use. A typical use case is described here: Firstly, functional connectivity (FC) maps were calculated in patients with Parkinson’s disease and controls based on resting-state fMRI. Subsequently, these maps were statistically compared between groups. This was followed by cluster-based inference with a voxel-level cluster-forming threshold and subsequent cluster-based thresholding correcting for multiple comparisons (MC). Clusters above this threshold were visualized in terms of few exemplary images [5]. Statistically similar approaches for fMRI data analyses are frequently applied in task-based fMRI [6] and coordinate based meta-analyses [7].

There has been a longstanding debate about adequate MC correction methods and thresholds (both on the voxel- and cluster-level) for such mass-univariate fMRI analyses given the statistical dependence of features (e.g. voxels) [6, 8–16]. This statistical dependence is caused by several factors including acquisition-related data smoothness and true biological interactions [12]. The issues of thresholding and MC correction interact with the sample sizes of a majority of current fMRI studies [17]. In this setting there is a risk of both false negative findings [13, 16, 18] and that published findings are false positive [13, 19]. In-depth criticisms of mass-univariate testing followed by MC correction in neuroimaging have been published [6, 20, 21]. See also a more general critical discussion about thresholds for statistical significance testing [22, 23] and an outlook on the more general scientific and clinical context in the final section of this article.

It has been argued that thresholding—though seemingly straightforward for statistical rigor—can lead to selective reporting and interpretation of for example few activation peaks passing a threshold while ignoring a wide array of subthreshold effects representing the potential true biological complexity of the neural processes of interest [16, 20, 24]. This has been referred to as the “localizationist bias” of such analyses [13, 25]. Such selective reporting might contribute to the heterogeneity and partial contradiction of fMRI results even from studies with similar research questions or analysis pipelines [6, 20, 26, 27]. It might also lead to false assumptions about the absence of an effect, when it does not pass the statistical threshold [28–30].

To alleviate this problem, it has thus been suggested that thresholded results could either be supplemented by suitable representations of potential subthreshold effects or that statistical approaches could be used which do not rely on thresholding of mass-univariate tests. In the following paragraphs we will give an overview of such established approaches as well as examples of newly emerging and related approaches. The focus of this review article is on scientific applications of fMRI.

## Supplementary Reporting of Unthresholded Result Maps

**Fig. 1:**
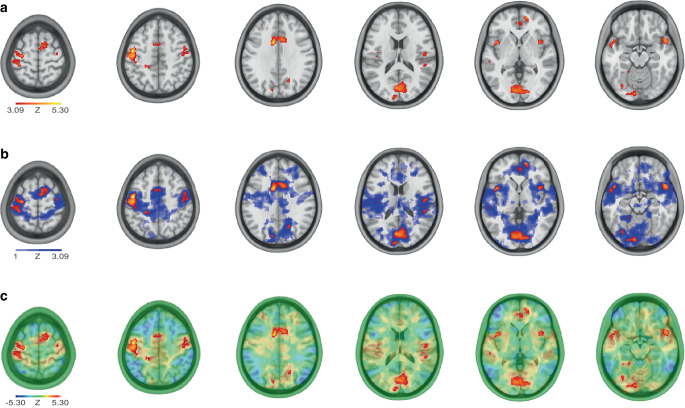
Exemplary illustrations incorporating subthreshold results based on an unthresholded Z‑map of group differences in physics problem solving-related brain networks. **a** Typical thresholded results map (voxel-wise threshold Z > 3.09, for illustration purposes only). **b** Thresholded results overlaid on a partially transparent representation of thresholded results with other Z‑thresholds (transparency increasing with decreasing Z‑threshold). **c** Thresholded results overlaid on a partially transparent bidirectional unthresholded Z‑map (Z-values represented by color coding)

Visualizations of sub-threshold results within research articles can include showing either unthresholded maps alongside thresholded results or combined figures. Combined figures can for example use different color scales or partial transparency [27]. This has also been elaborated on in more general articles on data visualization in neuroscience [28, 31]. Visualization examples are presented in Fig. 1 based on publicly shared (https://identifiers.org/neurovault.image:111654) unthresholded results from [32]. This approach can be extended to surface representations [33]. Such presentations of unthresholded maps can support interpretation of results, e.g. by highlighting underlying patterns such as those resembling functional networks [34], biological effects outside the areas of interest in an analysis, such as vascular patterns, areas with increased physiological pulsations or artefacts [35]. An example of figures containing unthresholded maps is the presentation of activation-likelihood estimation values and the comparison of results with unthresholded maps of temporally-independent functional modes in a meta-analysis of resting-state fMRI in depression [36]. There are today uncountable further application examples of such figures in the fMRI literature, however still only in a minority of fMRI articles reporting whole brain mapping results (authors’ impression). It has been recommended to avoid fixed thresholds of activation maps in clinical presurgical fMRI [37], a scenario mandating control for false negative results [38, 39]. As a quantitative alternative to unthresholded viewing, an adaptive thresholding approach taking the signal-to-noise ratio (SNR) into account has been suggested for such single subject task-based fMRI analyses [40]. As further clinical neuroimaging analogies, data such as those obtained from computed tomography perfusion in stroke [41] or beta amyloid positron emission tomography [42] are usually viewed or analysed without fixed thresholding.

In order to provide more information than typical main article figures, more detailed maps (e.g. tightly spaced axial slices) can be published as supplementary figures or 3D neuroimaging files (e.g. NIfTI). Sharing such representations has also been recommended in the *Committee on Best Practice in Data Analysis and Sharing* (COBIDAS) report on MRI [43]. An alternative to submitting unthresholded maps of statistical results with the article is submitting them to a dedicated repository, i.e. NeuroVault [44], which facilitates meta-analytical modeling based on such data representations [7, 45]. References from figure legends to unthresholded maps shared online might stimulate readers to further explore the underlying data. Another potential use of such shared unthresholded results is a direct comparison with e.g. new results or functional network maps by spatial cross correlation. Spatial cross correlation of unthresholded maps has, for example, been used to compare meta-analytical maps of task fMRI activations across studies with resting state network representations [46].

As a limitation, unthresholded maps of statistical results such as T‑, Z‑ or p‑maps can misrepresent the true size of the underlying effect. It has thus been suggested to supplement fMRI results with unthresholded effect size representations [47], confidence intervals [30] and measures of inter-subject variability [48]. For task-based fMRI the underlying effects can be further described based on properties of local hemodynamic response functions (HRF) estimated from the data [49–51]. Finite impulse response models [52] are an example of such HRF estimation methods. Possibilities for presenting such HRF results range from unthresholded mapping of quantitative HRF properties to detailed local depiction of HRF curves and quantitative HRF comparison based on a‑priori knowledge of the brain regions involved in the processing of a task [49]. This HRF evaluation framework can provide additional insight by providing a more direct link between physiology and statistical results. It can thus help better understand results of conventional analyses of fMRI data, both above and below conventional statistical thresholds. Another limitation is that visually interpreting unthresholded maps introduces elements of exploratory data analysis into an analysis formally guided by hypothesis testing. It can, however, be argued that whole brain fMRI analyses as discussed here (see region of interest analyses as an alternative [53]) even if testing highly specific hypotheses regarding e.g. group differences or task effects are often not region- or network-specific. They may thus already contain exploratory elements in the spatial domain.

## P-value Histograms and Higher Criticism

**Fig. 2:**
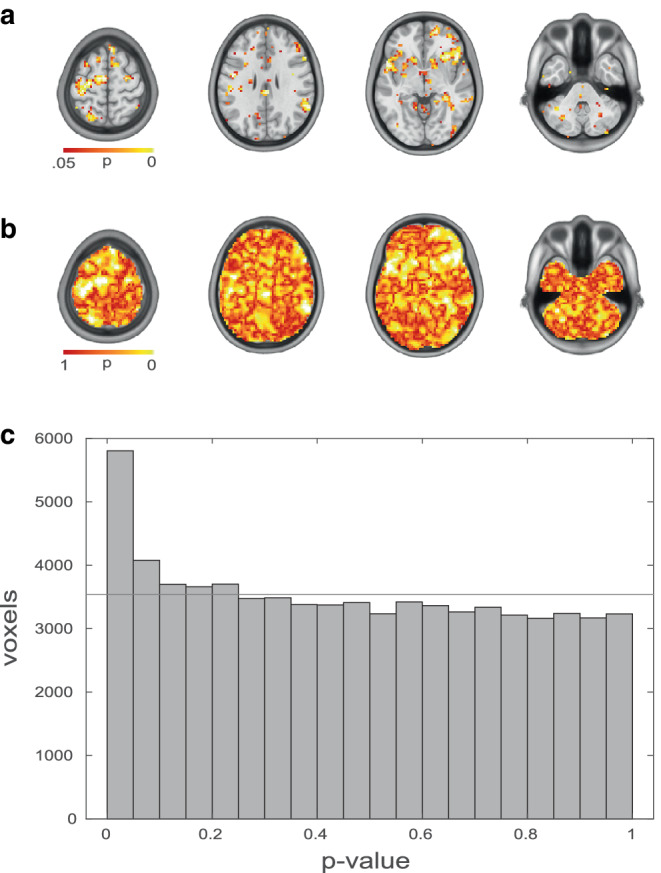
Example of a *p*-value distribution based on an unthresholded p‑map representing the main effect of pain predictive cues on brain responses to medium heat in a mediation analysis. **a** Typical representation of a thresholded results map (liberal voxel-wise threshold *p* < 0.05 chosen for illustration purposes only). **b** Unthresholded p‑map overlaid on an anatomical template. **c** *p*-value histogram based on all non-zero voxels. Under the null hypothesis (absence of an effect) an even distribution of *p*-values (horizontal line) would be expected. The overabundance of lower *p*-values indicates that there is an effect in line with the alternative hypothesis of the underlying voxel-wise statistical tests. The general distribution of *p*-values (e.g. no peaks at medium or high *p*-values) indicates that there are no gross deviations from the statistical assumptions of the underlying individual tests

Plotting the distribution of *p*-values has been introduced as a sanity-check for mass-univariate analyses, since it can help identify issues e.g. with deviations from assumptions of the underlying tests or a lack of statistical power to detect effects despite substantial i.e. subthreshold information content in the data [54]. For an intuitive more detailed introduction to *p*-value histogram interpretation see: https://web.archive.org/web/20230101002916/http://varianceexplained.org/statistics/interpreting-pvalue-histogram/. Figure 2 based on publicly shared (https://identifiers.org/neurovault.image:785533) unthresholded results from [55] depicts the typical behavior in a mass-univariate analysis with information contained in the underlying data. This information content is represented by an excess of small *p*-values, i.e. deviating from an even distribution expected under the null-hypothesis [54]. The example in Fig. 2 illustrates the importance of sub-threshold effects more generally: Deviations from the even distribution are not limited to the histogram bin with the lowest *p*-values but can exhibit a gradual descent (e.g. the number of *p*-values in the second and third lowest histogram bin also exceeding the expected average number). This visually highlights the notion that there is further information contained in fMRI data despite the fact that individual pieces of information cannot be identified with sufficient reliability by thresholded representations. Examples of reporting *p*-value histograms in fMRI research is the assessment of underlying information content in a diagnostic classification study in depression which resulted only in low diagnostic accuracies in a relatively heterogeneous sample [56] and the assessment of individual subjects’ contributions to significance in task-based fMRI [57].

In simplified terms the Higher Criticism (HC) approach [58] takes the rationale of *p*-value histogram interpretation a step further: It not only visually interprets, but explicitly tests, whether there is an excess of low *p*-values [54]. The HC approach has been further refined [59] and assessed [60, 61]. HC can detect, “that” there are effects in a large number of univariate tests while not identifying “which” exact effects are present [58, 62]. It has thus been widely used to detect the presence of rare and weak effects [58] in several areas of high dimensional data analysis such as genetics [58] and economics [63]. Potential applications in fMRI research could be supplementary analyses: One might explore whether there are effects of interest within a region of interest or a functional brain network when conventional MC correction of mass-univariate testing fails to detect such effects in individual voxels or pairwise connections. It could also be conceived as a step-down approach, looking at larger systems or areas within the brain prior to a targeted use of conventional approaches as post hoc tests. HC has only recently been introduced to fMRI: A study by Gerlach et al. aimed to identify FC alterations associated with worry in older subjects. The authors opted for an HC-based second-level analysis based on 22,366 regression coefficients because they expected only rare and weak effects. Based on the HC approach they could reject the global null hypothesis that resting state FC has no association with worry. They carried out an iterative follow-up analysis to identify the individual FC features most likely contributing to this result by ranking their importance [64]. Further applications of HC in fMRI have since been reported [65–68]. One of these studies includes a more detailed introduction and methodological discussion of applying HC to FC [66]. Furthermore, network contingency analysis of functional connectivity group effects follows a similar rationale with a count- and permutation-based assessment of an excess of low *p*-values within segregated parts of the functional connectome [69].

Visualisations of unthresholded *p*-value distributions are established in high-throughput analyses outside functional neuroimaging: Genome-wide association studies (GWAS) results are usually visualized in “Manhattan plots” displaying the −log_10_(*p*-value) of each single-nucleotide polymorphism SNP [70]. Manhattan plots thus share similarities with both unthresholded *p*-value maps and *p*-value histograms. Notably, discussions about optimal thresholds, correction methods and inclusion of subthreshold effects for GWAS [71, 72] conceptually mirror discussions about thresholding in fMRI: The typically applied genome-wide significance threshold (*p* < 5 × 10^−8^) is considered to be rather stringent to foster reproducibility. Yet subsequently, a substantial proportion of additional loci only identified by relaxed *p*-value thresholds are potential true positives [72]. There is a potential for using Manhattan plots in neuroimaging beyond GWAS: An example is the visualisation of assocations between clinical variables and imaging-derived phenotypes in the UK biobank [73, 74].

A potential limitation of *p*-value histogram analysis is the assumption of statistical independence of the underlying observations [54, 68]. It has however been argued, that this can be negligible in cases of typical histogram behavior [54], which mandates their visual assessment and fully reporting them. A modified HC approach has been suggested which explicitly allows for arbitrary correlation structures among features [75]. More experience needs to be gained with the application of HC to fMRI data before its potential can be fully assessed.

## Multivariate Analyses

Many of the limitations of conventional fMRI analyses described above are based on mass univariate testing itself [6, 20]. It can thus be an alternative to opt for multivariate analysis techniques which incorporate information across many features (e.g. voxels), thus also making use of information falling below threshold in mass-univariate analyses. Such multivariate statistical approaches have been widely used for fMRI data analysis [76]. Nomenclature of multivariate approaches in fMRI research varies between contexts. We aim to provide examples of some frequently used approaches without aiming for a comprehensive overview.

Most multivariate analyses of fMRI adopt either supervised or unsupervised machine learning techniques integrating information from multiple (e.g. voxel- or connection-wise) features [76, 77]. In supervised learning, decision rules (i.e. classifiers) are learned based on labelled training data. The models aim at assigning new data to previously known categories [77–79]. This approach has been popular in the context of diagnostic modelling for psychiatric disorders [80–84]. On the other hand, unsupervised learning methods aim to identify previously unknown structure in unlabeled training data [77–79]. Unsupervised learning has have gained considerable attention in terms of multivariate subgrouping attempts in neuroimaging research on mental disorders [85–87]. For multivariate analysis approaches in individual subjects or groups aiming at elucidating cognitive processes the term multivoxel pattern analysis (MVPA) is frequently used. MVPA typically aims to explore the representational content of fMRI data [76]. Examples are decoding with supervised learning [88] or representational similarity analysis which can incorporate unsupervised learning methods [89]. Exploratory model-free analyses such as independent component analysis (ICA) belong to this field in a wider sense. They have for example been used to identify functional networks in resting-state fMRI data [90]. Resulting unthresholded network maps can be used in group comparison analyses with methods such as dual regression [91].

A conceptual limitation of multivariate approaches is that most of them pursue different goals compared with mass-univariate analysis as outlined in the introduction. They can thus not be a direct replacement for studies which mainly map brain activation patterns. Interpretability of parameter maps derived from these methods is limited, but interpretable machine learning for fMRI is evolving [92–94]. Multivariate analyses need thorough optimization and validation approaches [76, 95]. Such methods pose alternative challenges for statistical significance testing, including the need for computationally intensive permutation testing in many validation scenarios [96]. To avoid problems with overly high dimensionality of fMRI datasets, some form of feature set reduction often involving some kind of thresholding is usually applied [97].

## Dedicated Bayesian Analysis Approaches

Limitations of thresholding are mostly encountered in the setting of classical frequentist statistical approaches. Bayesian approaches to fMRI analysis have been introduced as potential remedies for limitations of such classical analyses. Thresholding-related issues also apply to many Bayesian approaches. It has, however, been highlighted, that the information to be thresholded (e.g. the true positive rate) can differ from classical hypothesis testing [98]. Another Bayesian approach supplementing classical hypothesis testing is using Bayes factors for null effect assessment [99]. A comprehensive review of Bayesian neuroimaging analyses is not intended here.

Recently, a Bayesian multilevel modelling approach has been suggested to address limitations of mass-univariate testing with MC correction with reporting of thresholded results. It incorporates the cross-space information contained in fMRI data into coherent models and involves reporting of effect sizes and uncertainties [20]. This approach is currently seeing initial applications [100].

Main limitations of Bayesian approaches addressing the issue of dichotomization in classical hypothesis testing approaches is their hitherto limited use. Thus, more experience has to be gained.

## Outlook: fMRI Subthreshold Effects in a Wider Context

Ultimately, the current and future role of subthreshold effects in neuroimaging will also depend on consensus in the scientific community including preferences of readers, journals and funding bodies. Given concerns for poor reproducibility of scientific results, well thought ought arguments have been put forth to adopt more rigorous statistical significance thresholds [22]. Similarly, reporting and interpreting “marginally non-significant results” or “trends towards significance” have been discouraged [101]. However, the latter arguments have often been discussed in the context of providing e.g. confidence intervals which also convey information about potential subthreshold effects and underlying uncertainties [102]. To date, no broad consensus on these more general issues appears to have been reached.

Regarding this intention for scientific rigor, it should also be considered that while some of the approaches to subthreshold effects rely on quantitative and objective measures (e.g. multivariate analysis, HC, Bayesian approaches), others rely on visual or exploratory data analyses (e.g. visualisation of unthresholded maps, *p*-value histogram interpretation). In order to better assess the reliability of results from the latter approaches, fMRI data quality metrics [103, 104] can additionally be taken into account. These include head motion estimates [105, 106] particularly important for resting-state fMRI, the signal-to-noise ratio (SNR) [107] relevant for fMRI in general and the contrast-to-noise-ratio (CNR) [107, 108], mainly for task-based fMRI. The SNR affects the t‑statistic of voxel-wise tests [47] and in some definitions CNR estimates contain information about the effect size [107].

Sub-threshold effects as discussed in this article apply to high-dimensional neuroimaging representations such as those covering the whole brain. Efforts to improve the rigor of hypothesis-driven research include registered reports [109]. It could be conceived that they might lead to a stronger focus on testing hypotheses in lower-dimensional representations of neuroimaging data (e.g. in smaller regions [110] or individual networks). This could potentially alleviate some of the drawbacks of mass-univariate testing.

This article focuses on scientific applications of fMRI with few additional examples from clinical neuroimaging applications. While scientific group studies usually try to avoid false positive results, clinical applications in single patients often aim to avoid false negative results, i.e. not miss relevant areas of activation [38, 39]. The applicability of the advanced statistical models (i.e. Bayesian or HC-based) presented here and their derivatives in presurgical fMRI needs to be assessed in future studies.

## Conclusion

Mass-univariate analyses with thresholded presentation of results remains a mainstay of fMRI research. Among other limitations, this approach can lead to overly-selective conclusions based only on results passing a threshold, i.e., the tip of the iceberg. Different ways for supplementary data representations of subthreshold information have been suggested including repositories and improved visualization. We do not intend to call for lax multiple comparison correction thresholds. However, knowledge about e.g., underlying activation patterns or noise levels might improve interpretation and comparability of statistically significant above-threshold findings. It might also help better interpret the absence of findings in some of such studies. Thus, in our opinion supplementary reporting of subthreshold results is still widely underrepresented in the neuroimaging literature. Further analysis approaches are explicitly designed to leverage wider arrays of information which might not pass thresholds in conventional analyses. Among these are well-established multivariate analyses. This article also highlights novel data analysis approaches hitherto rarely used in neuroimaging: Examples are Higher Criticism for detecting rare and weak effects and dedicated Bayesian techniques. We believe that further research is warranted to assess the potential of such approaches to supplement conventional neuroimaging analyses and help improve interpretability and reliability in this field.

## Data Availability

Brain images were created with Mango (https://mangoviewer.com/mango.html, RRID: SCR_009603) based on unthresholded result maps derived from NeuroVault (https://identifiers.org/neurovault.image:111654 and https://identifiers.org/neurovault.image:785533). We thank the authors of the original studies [32, 55] for publicly sharing these maps.
